# Immunophenotyping Reveals the Diversity of Human Dental Pulp Mesenchymal Stromal Cells *In vivo* and Their Evolution upon *In vitro* Amplification

**DOI:** 10.3389/fphys.2016.00512

**Published:** 2016-11-08

**Authors:** Maxime Ducret, Hugo Fabre, Olivier Degoul, Gianluigi Atzeni, Colin McGuckin, Nico Forraz, Frédéric Mallein-Gerin, Emeline Perrier-Groult, Brigitte Alliot-Licht, Jean-Christophe Farges

**Affiliations:** ^1^Laboratoire de Biologie Tissulaire et Ingénierie Thérapeutique, UMR5305 Centre National de la Recherche Scientifique/Université Lyon 1, UMS3444 BioSciences Gerland-Lyon SudLyon, France; ^2^Faculté d'Odontologie, Université de Lyon, Université Lyon 1Lyon, France; ^3^Hospices Civils de Lyon, Service d'OdontologieLyon, France; ^4^Laboratory of Regenerative Technologies, Department of Biomedical Engineering, University of BaselBasel, Switzerland; ^5^CTI-BIOTECH, Cell Therapy Research InstituteMeyzieu, France; ^6^Institut National De La Santé Et De La Recherche Médicale UMR1064, Faculté d'Odontologie, Centre de Recherche en Transplantation et Immunologie, Université de NantesNantes, France

**Keywords:** human dental pulp, mesenchymal stromal cells, stem cell markers, CD56, CD146, immunophenotyping, regenerative dentistry, cell-based medicinal products

## Abstract

Mesenchymal stromal/stem cells (MSCs) from human dental pulp (DP) can be expanded *in vitro* for cell-based and regenerative dentistry therapeutic purposes. However, their heterogeneity may be a hurdle to the achievement of reproducible and predictable therapeutic outcomes. To get a better knowledge about this heterogeneity, we designed a flow cytometric strategy to analyze the phenotype of DP cells *in vivo* and upon *in vitro* expansion with stem cell markers. We focused on the CD31^−^ cell population to exclude endothelial and leukocytic cells. Results showed that the *in vivo* CD31^−^ DP cell population contained 1.4% of CD56^+^, 1.5% of CD146^+^, 2.4% of CD271^+^ and 6.3% of MSCA-1^+^ cells but very few Stro-1^+^ cells (≤ 1%). CD56^+^, CD146^+^, CD271^+^, and MSCA-1^+^ cell subpopulations expressed various levels of these markers. CD146^+^MSCA-1^+^, CD271^+^MSCA-1^+^, and CD146^+^CD271^+^ cells were the most abundant DP-MSC populations. Analysis of DP-MSCs expanded *in vitro* with a medicinal manufacturing approach showed that CD146 was expressed by about 50% of CD56^+^, CD271^+^, MSCA-1^+^, and Stro-1^+^ cells, and MSCA-1 by 15–30% of CD56^+^, CD146^+^, CD271^+^, and Stro-1^+^ cells. These ratios remained stable with passages. CD271 and Stro-1 were expressed by <1% of the expanded cell populations. Interestingly, the percentage of CD56^+^ cells strongly increased from P1 (25%) to P4 (80%) both in all sub-populations studied. CD146^+^CD56^+^, MSCA-1^+^CD56^+^, and CD146^+^MSCA-1^+^ cells were the most abundant DP-MSCs at the end of P4. These results established that DP-MSCs constitute a heterogeneous mixture of cells in pulp tissue *in vivo* and in culture, and that their phenotype is modified upon *in vitro* expansion. Further studies are needed to determine whether co-expression of specific MSC markers confers DP cells specific properties that could be used for the regeneration of human tissues, including the dental pulp, with standardized cell-based medicinal products.

## Introduction

Dental and craniofacial research currently explores a variety of cell-based and tissue engineering protocols to be used as alternatives to classical therapies that aimed at repairing/regenerating damaged tissues (Ducret et al., 2015a; Gorin et al., 2016). In particular, studies have demonstrated that mesenchymal stromal/stem cells (MSCs) are suitable to these protocols because of their high expansion ability and differentiation potential both in culture and animal models. MSC-based therapies have already been applied in the clinics and mainly consist of administration of cells, alone, or together with scaffolds, to damaged, or pathological sites (Pagella et al., 2015). Bone marrow (BM), Wharton's jelly and adipose tissue are conventional sources of MSCs, but owing to the easy tooth access and the low morbidity of tooth collection protocols, dental pulp (DP) previously emerged as an valuable source of MSCs for tissue engineering-based therapies (Huang et al., 2009; Harrington et al., 2014). The DP is the loose connective tissue located in the center of the tooth. Its main cellular components are neural crest-derived mesenchymal cells that mostly reside in the tissue in the form of undifferentiated mesenchymal cells, fibroblasts, and highly differentiated dentin-forming odontoblasts. Pioneering studies of Gronthos et al. (2000) demonstrated that undifferentiated DP cells include a small population—less than 5% of cells—that possess phenotypic features of MSCs and are able to form a pulp-dentin complex upon engraftment *in vivo* in immunodeficient mice (Gronthos et al., 2000). DP-MSCs mostly reside in perivascular stem cell niches that provide cells a highly regulated microenvironment instructing them to remain quiescent and preventing them to proliferate, differentiate, or undergo apoptosis (Moore and Lemischka, 2006; Mitsiadis et al., 2007; Pagella et al., 2015). Perivascular localization of DP-MSCs was ascertained by the fact that a large proportion (more than 60%) of clonogenic DP-MSCs were present in the pericyte fraction and by their expression of specific pericyte and smooth muscle cell markers (Shi and Gronthos, 2003; Alliot-Licht et al., 2005; Lopez-Cazaux et al., 2006). Since then, several authors have reported the existence, in the DP, of other MSC populations whose proliferation and differentiation potentials are similar (Iohara et al., 2006; Sonoyama et al., 2008; Huang et al., 2009; Kawashima, 2012; Lv et al., 2014; Mayo et al., 2014). However, it remains unclear if these populations also include sub-populations which may differ in their self-renewal properties, lineage commitment, and differentiation capabilities toward specific phenotypes such as pulp fibroblasts and odontoblasts. This knowledge is however of paramount importance since cell heterogeneity may be a hurdle to the achievement of reproducible and predictable therapeutic outcomes.

Although no definitive MSC markers have been identified so far (Lv et al., 2014), DP-MSC populations have been characterized mainly on the basis of the expression of cell surface molecules including the bone marrow cell membrane antigen Stro-1 (Gronthos et al., 2000; Alliot-Licht et al., 2005), the melanoma cell adhesion molecule MCAM/CD146 (a marker of perivascular MSCs; Shi and Gronthos, 2003; Lv et al., 2014; Harkness et al., 2016), the low affinity nerve growth factor receptor p75NTR/CD271 (Waddington et al., 2009; Lv et al., 2014; Alvarez et al., 2015; Tomlinson et al., 2016), the mesenchymal stem cell antigen MSCA-1 (also known as TNAP/Tissue Non-Specific Alkaline Phosphatase; Sobiesiak et al., 2010; Tomlinson et al., 2015), and the neural cell adhesion molecule NCAM/CD56 (a marker of neural and muscular MSC populations; Battula et al., 2009; Sobiesiak et al., 2010; Bonnamain et al., 2013; Lv et al., 2014). We recently isolated and easily amplified in culture a population of MSCs from the DP of human developing third molars with a medicinal manufacturing approach (Ducret et al., 2015b). We showed by using flow cytometry that all cells of this population expressed the mesenchymal cell markers CD10, CD13, CD29, CD44, CD90, CD105, and CD166 *in vitro*, but did not express the hematopoietic markers CD14, CD34, CD45, CD79a, and HLA-DR or the endothelial cell/leucocyte marker CD31 (Ducret et al., 2015a,b; Ducret et al., 2016). Stro-1 and CD271 were expressed by a very low number (≤ 1%) of cultured DP-MSCs, whereas CD146 and MSCA-1 were expressed by about 40 and 15% of DP-MSCs, respectively. These results suggested that the DP-MSC population was heterogeneous and may contain several populations with different phenotypic and biological properties. The objective of this study was to identify more precisely the MSC populations present in the human DP *in vivo* and the DP-MSC populations that can be isolated and expanded up to four passages with our GMP approach. We analyzed with flow cytometry the expression of CD56, CD146, CD271, MSCA-1, and Stro-1 on CD31^−^ DP cells to exclude endothelial and leukocytic cells that may express some of the above markers although being not MSCs.

## Materials and methods

### Isolation and amplification of human dental pulp cells

Healthy impacted human third molars were collected from donors aged 13–17 years with informed consent of the patients and their parents, in accordance with the recommendations of the World Medical Association's Declaration of Helsinki and following a protocol approved by the French Ministry of Higher Education and Research (CODECOH: DC-2014-2325). Dental pulps from teeth between Nolla developmental stages 5 (crown almost completed) and 7 (one third root completed) were gently extirpated from pulp cavities and cut into fragments of about 0.5–2 mm^3^. For *in vivo* immunophenotyping analysis, pulp fragments were digested in a mixture of 3 mg/mL collagenase type I (Calbiochem, San Diego, CA, USA) and 4 mg/mL dispase (Roche Diagnostics, Meylan, France) at 37°C for 1 h. The cell suspension was then washed twice with sterile PBS, passed through a 100-μm nylon mesh filter, and resuspended in phosphate buffered saline (PBS). For *in vitro* immunophenotyping analysis, fragments were treated as previously described (Ducret et al., 2015b). Briefly, they were seeded as explants on dishes pre-coated with human placental collagens I and III (ABCellBio, Paris, France), and then cultured in the chemically defined culture medium SPE-IV/EBM (ABCellBio) supplemented with 100 IU/mL penicillin and 100 *μ*g/mL streptomycin (Life Technologies, Saint Aubin, France). DP-MSCs outgrowing from the explants were passaged four times with the xeno-free recombinant protease TrypLe® Select 1X (Life Technologies) for amplification.

### Multiparametric flow cytometry

**Figure 1:**
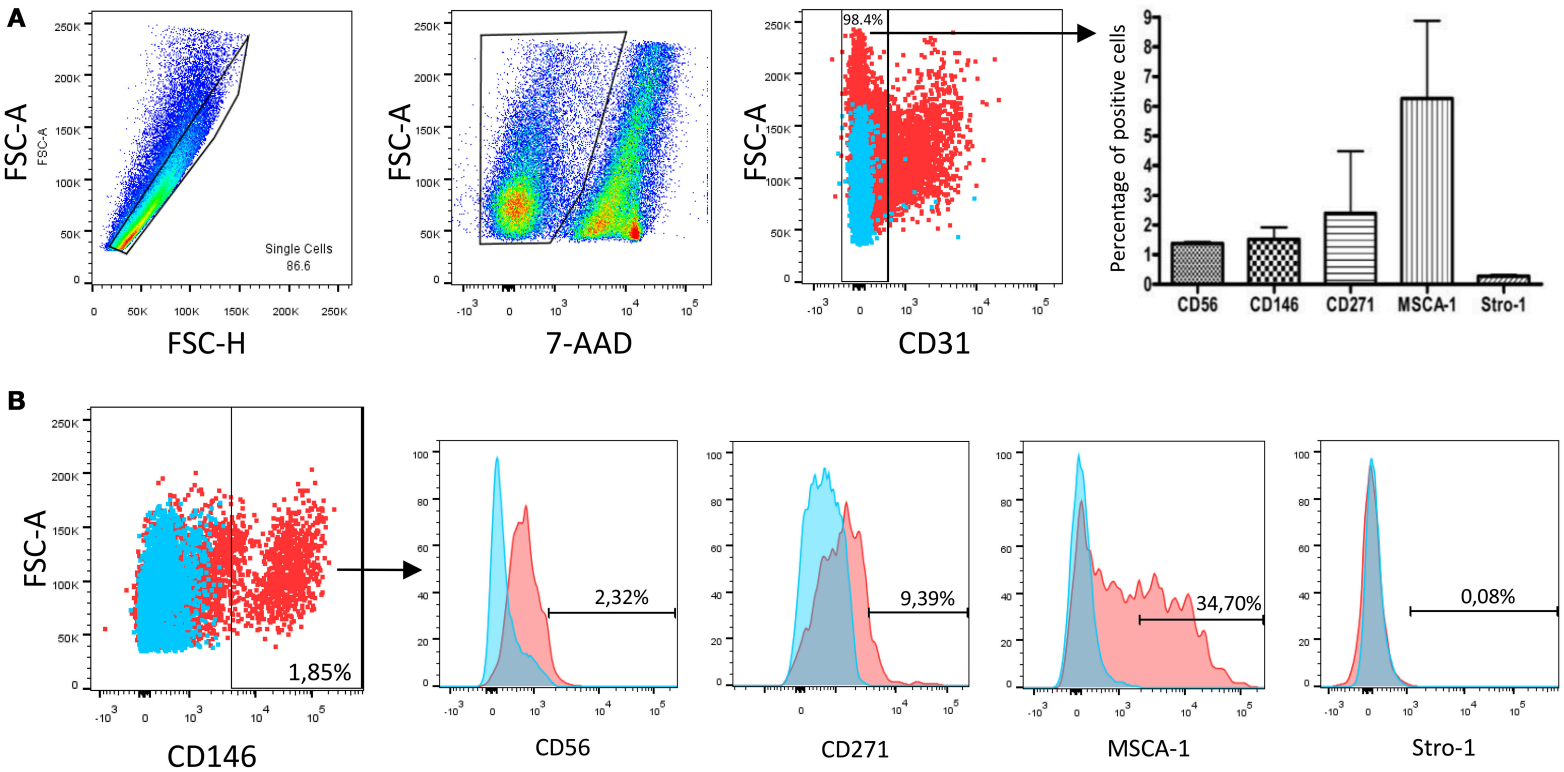
**Immunophenotypic characterization of human dental pulp mesenchymal stromal cells ***in vivo***. (A)** Flow cytometry gating strategy used for the removal of debris, doublets, dead cells and CD31^+^ cells. The CD31^−^ cell population obtained after enzymatic digestion of the whole dental pulp represented 98.4% of all recovered DP cells. It contained 1.38 ± 0.04% of CD56^+^ cells, 1.52 ± 0.40% of CD146^+^ cells, 2.39 ± 2.09% of CD271^+^ cells, 6.26 ± 2.62% of MSCA-1^+^, but very few Stro-1^+^ cells (0.27 ± 0.05%). Mean values ± standard deviation obtained from 3 dental pulps from different patients. **(B)** Proportion of CD31^−^ DP cells stained with the CD146 antibody and percentages of CD56, CD271, MSCA-1, and Stro-1 positive cells in the CD146^+^ population. Data shown are from one representative patient. *n* = 3.

The staining panel was designed by using 6 fluorochrome-conjugated antibodies, and the nucleic acid dye 7AAD (7-Amino-Actinomycin D, BD-Biosciences) was used for the exclusion of non-viable cells (Table 1). Fluorescence Minus One (FMO) controls were used in combination with isotype controls by replacing the missing antibody in every FMO control tube with the corresponding isotype control. This method enabled both the visualization of staining issues (isotype control) as well as gating boundaries (FMO control) without the need for multiple tubes, thus saving time in sample preparation while requiring fewer cells. Unstained controls were also used to check background- and cell auto-fluorescence. Cell suspensions were prepared by incubating 1.10^7^ cells/mL in staining buffer containing 1% bovine serum albumin (BD Pharmingen, Le Pont de Claix, France). Hundred *μ*L of cell suspension were then added to each of the eight different antibodies combinations (Table 2) and incubation was performed for 25 min at 4°C in the dark. Stained cells were washed with BD Lyse Wash Assistant (BD Biosciences) to maximize cell viability and prevent cell adhesion to the staining tube. Samples were kept on ice and analyzed within 2 h of processing after 10 min of incubation with 7-AAD. The gating strategy was as follows: A primary gate was placed on the area vs. height signal of the forward scatter (FSC-A/FSC-H) dot plot to discriminate for doublets and clumps, and a Boolean gate was then set on the 7-AAD^neg^ cells to enable the analysis of the viable single cell population. CD31^+^ (endothelial and leukocytic) cells were excluded from the analysis by setting a gate that encompassed 99.9% of the events in the APC-Cy7 channel of the FMO control tube 6 (Table 2, Figure 1A). The single cell population was identified by defining the gated population on a side scatter area signal vs. a forward scatter area (SSC-A/FSC-A) signal dot plot. The target number of acquired events for each tube of the panel was 3.10^5^ for *in vitro* and 5.10^4^ for *in vivo* immunophenotyping. This target was set to 1.10^5^ events for unstained, isotype-FMO and compensation controls. Data were acquired as uncompensated events and recorded as FCS 3.0 files. Analysis and compensation was performed using the FlowJo vX software (TreeStar Inc., Ashland, OR, USA).

**Table 1:** **Fluorochrome-conjugated monoclonal antibodies used for immunophenotypic analysis**.

**Target**	**Fluorochrome**	**Clone**	**Manufacturer**	**Reference**	**Isotype**	**Reference**
CD31	APC-Cy7	WM59	Biolegend	303120	Mouse IgG1, k	400128
CD56	BV510	HCD56	Biolegend	318340	Mouse IgG1, k	562946
CD146	AF488	SHM-57	Biolegend	342008	Mouse IgG2a, k	400233
CD271	PE-Cy7	C40-1457	BD-Biosciences	562852	Mouse IgG1, k	557646
MSCA-1	APC	W8B2	Biolegend	327308	Mouse IgG1, k	400120
Stro-1	PE	IgM, l	Santa Cruz	sc-47733 PE	Mouse IgM, l	sc-2870

**Table 2:** **Fluorescence Minus One (FMO) control strategy**.

**FMO control tubes**							**Panel**
**Tube 1**	**Tube 2**	**Tube 3**	**Tube 4**	**Tube 5**	**Tube 6**	**Tube 7**	
Isotype	CD56	CD56	CD56	CD56	CD56	CD56	CD56
CD146	Isotype	CD146	CD146	CD146	CD146	CD146	CD146
Stro-1	Stro-1	Isotype	Stro-1	Stro-1	Stro-1	Stro-1	Stro-1
CD271	CD271	CD271	Isotype	CD271	CD271	CD271	CD271
MSCA-1	MSCA-1	MSCA-1	MSCA-1	Isotype	MSCA-1	MSCA-1	MSCA-1
CD31	CD31	CD31	CD31	CD31	Isotype	CD31	CD31
7AAD	7AAD	7AAD	7AAD	7AAD	7AAD	Empty	7AAD

### Statistical analysis

Values were presented as the mean ± standard deviation, and differences were analyzed using the Mann-Whitney *U*-test for nonparametric analysis. The number of independent samples from different donors (n) is indicated in figure legends. A *P* < 0.05 was signified with ^*^ and *P* < 0.01 with ^**^.

## Results

The human DP is well known to contain MSCs, but not sufficient information is available regarding the immunophenotypic profile of these cells. We therefore performed a flow cytometry analysis of DP cells collected following enzymatic digestion of the whole tissue. In this study, all *in vivo* and *in vitro* data were obtained following the same gating strategy (Figure 1A): debris, non-viable cells and doublets were first excluded, and then the analysis was performed on the CD31^−^ DP cell population. We found that this population represented 98.4% of living cells in the whole DP population and that it contained about 1.4% of CD56^+^ cells, 1.5% of CD146^+^ cells, 2.4% of CD271^+^ cells, 6.3% of MSCA-1^+^, but very few Stro-1^+^ cells (≤ 1%). In order to increase our knowledge of DP cells expressing these MSC markers, we next gated the CD56^+^, CD146^+^, CD271^+^, and MSCA-1^+^ cell populations and analyzed the expression of these four markers. An example is given in Figure 1B in the CD146^+^ cell population. Quantified data for all gated cell populations are reported in Table 3. They reveal that about 1.8, 4.6, and 6.4% of the CD56^+^ cell population expressed CD146, CD271 and MSCA-1, respectively, whereas 2.6, 11.5, and 35.8% of the CD146^+^ cell population expressed CD56, CD271, and MSCA-1, respectively. CD56 and Stro-1 were found in 3.5%, CD146 in 9.1% and MSCA-1 in 18% of CD271^+^ cells. CD56 was expressed in 1.4% of MSCA-1^+^ cells, whereas CD146 and CD271 were expressed by about 8 and 5.3% of the MSCA-1^+^ population. Very few cells (<1%) expressed Stro-1 in the gated CD56^+^, CD146^+^, and MSCA-1^+^ populations. The multiplication of the percentage of DP cells expressing one marker by the proportion of these cells expressing as second marker indicated that, in decreasing order, CD146^+^MSCA-1^+^, CD271^+^MSCA-1^+^, and CD146+CD271^+^ cells constitute the most abundant MSC populations in the human DP, each population representing between 0.1 and 0.5% of the total of CD31^−^ DP cells.

**Table 3:** **Percentage of cells expressing MSC surface markers in gated DP cell populations (***n*** = 3)**.

**Gated DP cell population**	**Surface markers**				
	**CD56**	**CD146**	**CD271**	**MSCA-1**	**Stro-1**
CD56^+^	−	1.78±0.3	4.6±1.5	6.4±2.9	0.7±0.2
CD146^+^	2.6±0.6	−	11.5±3.9	35.8±3.3	0.4±0.3
CD271^+^	3.5±1.5	9.1±5.3	−	18±10.5	1.5±0.6
MSCA-1^+^	1.4±0.3	8.0±2.9	5.5±2.8	−	0.6±0.2

**Figure 2:**
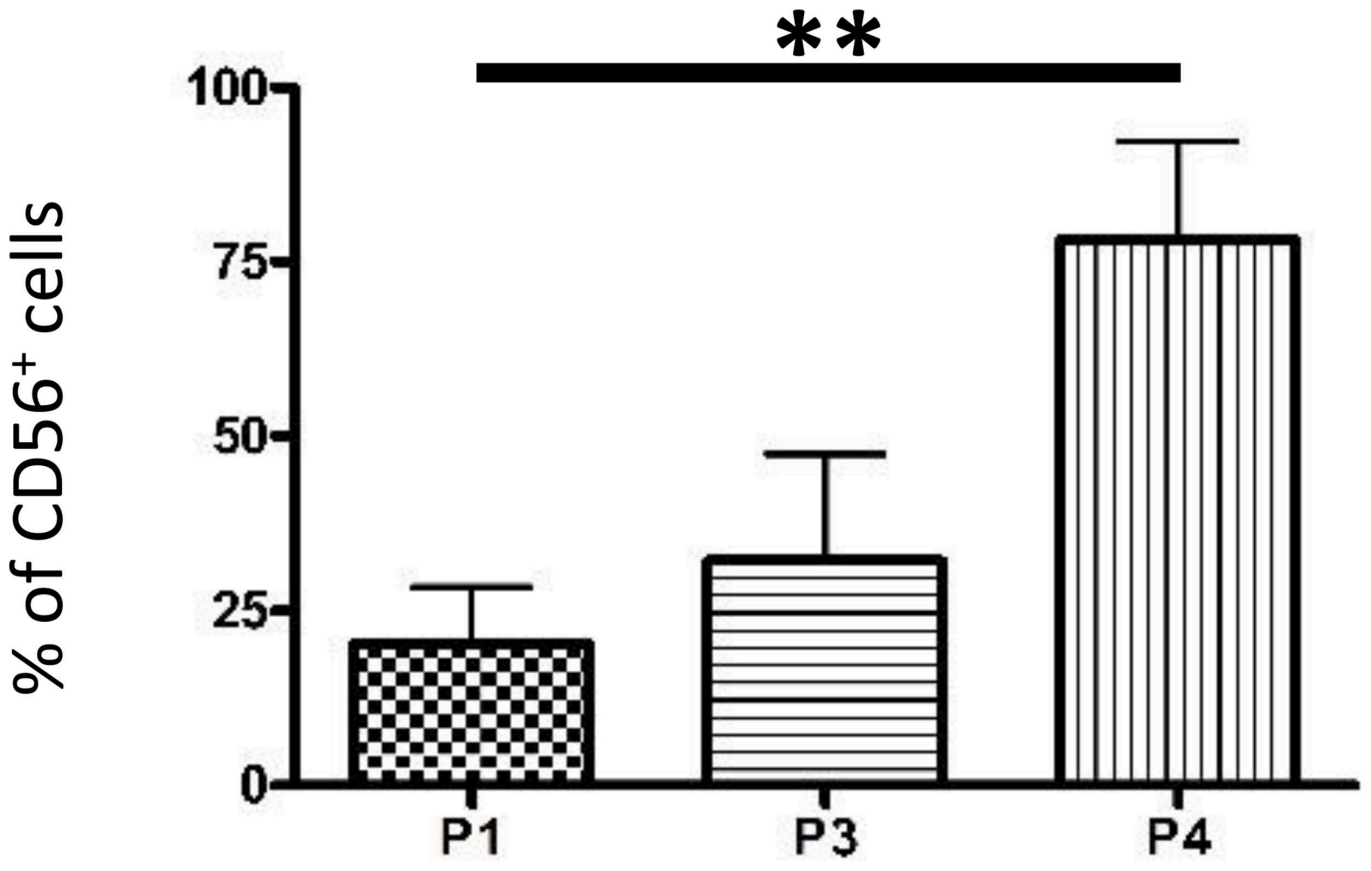
**Quantification of CD56^**+**^ DP-MSCs upon ***in vitro*** amplification**. Proportion of cells expressing CD56 in the whole DP-MSC population *in vitro* at the end of passages 1 (P1), 3 (P3), and 4 (P4). Error bars: mean ± SD. *n* = 5. ^**^ *P* < 0.01.

**Figure 3:**
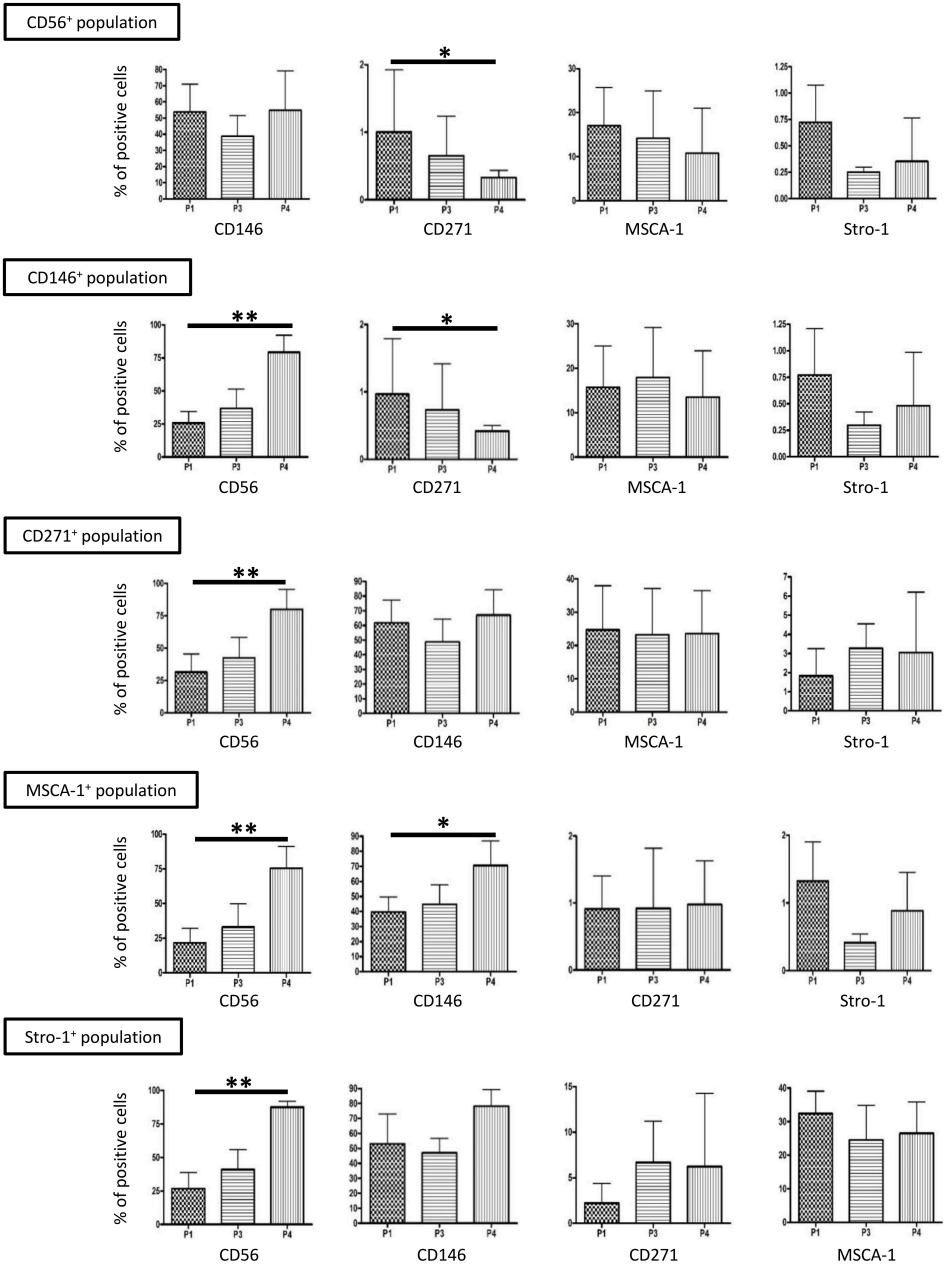
**Immunophenotypic analysis of CD56^**+**^, CD146^**+**^, CD271^**+**^, MSCA-1^**+**^, and Stro-1^**+**^ DP-MSC populations upon ***in vitro*** amplification**. MSC markers' expression in the gated populations during the amplification process. *P* < 0.05 was signified with ^*^ and <0.01 with ^**^. *n* = 5.

We previously showed that the whole DP-MSC population expanded *in vitro* in serum-free medium with a GMP approach did not express CD31, indicating that no endothelial or leukocytic cells were present in our cultures. A high amount of DP-MSCs was found to express CD146 (about 40%) and MSCA-1 (15%), but CD271 and Stro-1 were expressed by a very low number of cells (≤ 1%). These percentages remained unchanged during the culture time analyzed, i.e., from passage 1 (P1) to passage 4 (P4) (Ducret et al., 2015b). In the present study, we further observed that the percentage of CD56^+^ DP-MSCs in the whole cultured DP-MSC population, already high at P1 (about 25%), increased up to P4 to reach 80% (Figure 2). The percentage of CD56^+^ cells similarly increased in all the gated CD146^+^, CD271^+^, MSCA-1^+^, and Stro-1^+^ DP-MSC populations (Figure 3). CD146 was also expressed at high level, by about half the cells in the CD56^+^, CD271^+^, MSCA-1^+^, and Stro-1^+^ populations. This ratio remained stable with passages, except in the MSCA-1^+^ population in which CD146 expression increased significantly. The percentage of cells expressing CD271 was very weak, lower than 1%, in CD56^+^, CD146^+^, and MSCA-1^+^ cells, but was higher (≈5%) in the Stro-1^+^ population. A significant decrease of CD271 expressing cells was observed in the CD56^+^ and CD146^+^ populations. The percentage of cells expressing MSCA-1 was around 15–30% in gated cell populations and remained unchanged with passages. Finally, Stro-1 was expressed by a very weak percentage of cells (lower than 2%) in CD56^+^, CD146^+^, CD271^+^, and MSCA-1^+^ populations, and this percentage did not vary with the passages. Variations of MSC markers between passages 1 (P1) and 4 (P4) in the gated DP-MSC populations are summarized in Table 4. The multiplication of the percentage of DP-MSCs by the proportion of these cells expressing aseconds second marker indicated that CD146^+^CD56^+^ (about 10% of the total cell number), CD146^+^MSCA-1^+^ (≈6%), and MSCA-1^+^CD56^+^ (≈3%) cells were the most abundant DP-MSC populations at the end of P1 in our amplification conditions, whereas CD146^+^CD56^+^ (about 40%), MSCA-1^+^CD56^+^ (≈12%), and CD146^+^MSCA-1^+^ (≈8%) cells were the most abundant DP-MSC populations at the end of P4.

**Table 4:** **Variations of MSC markers between passages 1 (P1) and 4 (P4) in the gated DP-MSC populations**.

CD56	P1 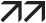 P4	CD146^+^ population CD271^+^ population MSCA-1^+^ population Stro-1^+^ population
CD146	P1 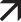 P4	MSCA-1^+^ population
CD271	P1 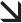 P4	CD56^+^ population CD146^+^ population

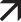
, 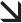 *P < 0.05*; 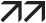 *P < 0.01*.

## Discussion

Human dental pulp (DP) contains quiescent mesenchymal stromal/stem cells (MSCs) which may be recruited from perivascular areas to repair or regenerate, once activated, the pulp/dentin tissue injured by dental caries, trauma or operative procedures (Huang et al., 2009). Owing to their strong capacities for proliferation and differentiation into DP specific cells such as odontoblasts, DP-MSCs have been isolated and expanded *in vitro* for cell-based and regenerative dentistry therapeutic purposes (La Noce et al., 2014). However, cells amplified *in vitro* are highly heterogeneous and comprise populations with different lineage commitment related to their *in vivo* environment (Lv et al., 2014). This heterogeneity is considered a hurdle to the achievement of reproducible and predictable therapeutic outcomes (Ishizaka et al., 2012). To gain more insight into DP-MSC heterogeneity, we quantified the number of DP cells expressing the mesenchymal stromal/stem cell markers CD56, CD146, CD271, MSCA-1, and Stro-1. We focused our study on CD31^−^ DP-MSCs to exclude cells that may express some of these markers although being not MSCs. Indeed, some endothelial cells are known to express CD146 and Stro-1, and natural killer cells and some lymphocytes may express CD56 (Shi and Gronthos, 2003; Woodfin et al., 2007; Crisan et al., 2008; Ge et al., 2013; Lv et al., 2014; Harkness et al., 2016; Melsen et al., 2016). Flow cytometry was recently used to characterize the various populations of immune cells present in the human DP with a strategy of progressive cell gating (Gaudin et al., 2015), and we designed in the present study a similar strategy to allow for the identification and quantification of DP-MSC subpopulations. We found that the CD31^−^ DP cell population, which represented more than 98% of the DP cells isolated with our protocol, contained about 1.4% of CD56^+^ cells, 1.5% of CD146^+^ cells, 2.4% of CD271^+^ cells, 6.3% of MSCA-1^+^, but very few Stro-1^+^ cells (<0.3%). CD146^+^ cells were previously identified by immunohistochemistry in the human DP (Shi and Gronthos, 2003; Alongi et al., 2010). More recently, Huang et al. (2014) reported, by using flow cytometry, the presence of 2% of CD146^+^ in the whole DP cell population. Our data indicate that CD146^+^ cells represent about 1.5% of the CD31^−^ DP cell fraction. This lower amount may be explained by the fact that we have excluded the CD31^+^CD146^+^ DP cells, which represent DP endothelial cells (Nakashima et al., 2009). Conversely, the percentage of CD271^+^ and MSCA-1^+^ cells was similar to that reported in other studies (Waddington et al., 2009; Alvarez et al., 2015; Tomlinson et al., 2015, 2016; Pan et al., 2016; Yasui et al., 2016). We next investigated whether the various DP-MSC subpopulations expressed another one of these markers. Analysis of the CD31^−^CD146^+^ and CD31^−^CD271^+^ DP cell populations indicated that they contain 35.8 and 18% of MSCA-1^+^ cells, respectively, but only a low amount of Stro-1^+^ cells. The proportion of CD271^+^ cells positive for CD146 and Stro-1 was about 9.1 and 1.5%, respectively, a percentage much lower than that recently reported in DPs from adult patients (Yasui et al., 2016). Although both studies are not directly comparable since these authors only considered CD90^+^CD271^+^ DP cells, differences between their results and ours might also be related to the age of the patients since the wisdom teeth we used were from younger, 13–17 year-old patients. Co-expression of CD146 and MSCA-1 was previously detected in perivascular cells from adipose and muscular tissues where it was particularly abundant in pericytes (Crisan et al., 2008). Since MSCA-1 was shown to be mainly and strongly expressed in the subodontoblast cell-rich zone in human teeth (Zweifler et al., 2014), it is possible that the CD146^+^MSCA-1^+^ cell population we identified in the human DP originated from pericytes localized in the subodontoblast perivascular region (Alliot-Licht et al., 2005). To our knowledge, we are the first to report the quantification of CD56^+^ cells in the human DP by flow cytometry. CD56 was previously localized by immunohistochemistry in the central region of the mouse molar DP, but no indication was given on the type of cells that expressed it (Obara and Takeda, 1993). More recently, CD56 was immunolocalized in Schwann cells surrounding perivascular nerve fibers in the DP of patients who had undergone head and neck radiotherapy (Faria et al., 2014). Since Schwann cells are able to generate MSCs then to give rise to pulp cells and dentin-producing odontoblasts in damaged adult mouse growing incisors (Kaukua et al., 2014), it is possible that the CD56^+^ cells we identified in the healthy human DP are part of the Schwann cell population, given the capacity of our cultured CD56^+^ DP-MSCs to differentiate into odontoblast-like cells upon specific induction (Ducret et al., 2015b).

Besides the heterogeneity of the MSC population isolated from the tissue, modification of cell immunophenotype during amplification is an additional factor of MSC diversity *in vitro* (Cournil-Henrionnet et al., 2008; Kozanoglu et al., 2009; Bonnamain et al., 2013). We previously showed that the proportion of CD146^+^, CD271^+^, MSCA-1^+^ and Stro-1^+^ cells did not change in the whole DP-MSC population with passages (Ducret et al., 2015b). We further demonstrate here that CD146 and MSCA-1 were co-expressed by the higher number of cells in each gated DP-MSC population (about 50% and 15–30%, respectively). A similar CD146^+^MSCA-1^+^ cell population, isolated from the human skeletal muscle and non-muscle tissues, was shown to easily differentiate into myogenic cells in culture and *in vivo* (Crisan et al., 2008). CD146 is a transmembrane glycoprotein which belongs to the immunoglobulin superfamily of cell adhesion molecules. Its engagement leads to CD146 association with the actin cytoskeleton and regulation of cell migration (Ouhtit et al., 2009). It is constitutively expressed by endothelial cells, pericytes, smooth muscle cells, some lymphocytes, and melanoma cells (Bardin et al., 2001). It is also an early marker of MSCs of diverse origins, including bone marrow, adipose tissue, umbilical cord and dental pulp (Wu et al., 2016). CD146 is preferentially detected in MSCs with high clonogenicity, multipotency and differentiation potential (Lv et al., 2014). In human DP, CD146 was immunolocalized in perivascular cell niches (Shi and Gronthos, 2003). We previously showed that DP-MSCs expanded in our culture conditions contain a high percentage (≈50%) of CD146-expressing cells, suggesting that they may contain a high amount of multipotent DP-MSCs. Their capacity to differentiate into osteo/odontoblasts, adipocytes, and chondrocytes (Ducret et al., 2015b; Fabre et al., in preparation) are in accordance with this hypothesis.

One major finding of our work is the progressive increase in the number of CD56-expressing DP-MSCs with passages *in vitro*. CD56 (also called Neural Cell Adhesion Molecule or NCAM) is a cell adhesion molecule which belongs to the superfamily of immunoglobulin receptors. It is widely expressed in the central nervous systems, in which it mediates several neuronal functions by controlling intercellular adhesion, neurite outgrowth, and cell migration, proliferation, survival and differentiation. These events are triggered by the homophilic interaction of CD56 molecules on adjacent cells as well as by the heterophilic binding of CD56 to other adhesion molecules, extracellular matrix components or cell surface receptors (Hinsby et al., 2004; Francavilla et al., 2007; Cavallaro and Dejana, 2011; Leshchyns'ka and Sytnyk, 2016). CD56 is also expressed by muscle-derived stem cells and natural killer cells and, interestingly, by cells migrating from the cephalic neural crest (Kolkova et al., 2000; Sinanan et al., 2004; Melsen et al., 2016). Upon *in vitro* expansion of DP-MSCs in our serum-free culture conditions, the proportion of CD56^+^ cells increased in the whole population, as well as in gated CD146^+^, CD271^+^, MSCA-1^+^, and Stro-1^+^ cells, from about 25% at the end of P1 to 80% at the end of P4. This increase was very similar in all subpopulations, indicating that it is not related to the expression of the other MSC markers tested. High proportions of CD56^+^ cells have been previously identified in cultures of human DP-MSCs, ranging from about 50–70% of the whole cell population (Degistirici et al., 2008; Bonnamain et al., 2013; Tomlinson et al., 2015), but we are the first to show the progressive increase in the number of CD56^+^ cells without the use of a neuro-inductive culture medium. This increase might be related to the selection of a specific CD56^+^ cell population in DP explants by our culture conditions, in relation to components present in the culture medium. Another possibility is that CD56^+^ DP-MSCs migrating from the DP explants possess high proliferative intrinsic capacities which allow them for progressively overriding the CD56^−^ DP-MSC populations. Since DP mesenchymal cells are of neural crest origin, several authors have suggested that cultures of DP-MSCs expanded *in vitro* contained a high proportion of cells of neural crest origin (Aurrekoetxea et al., 2015). Although a direct link between neural crest-derived DP-MSCs and the neural crest marker CD56 has not been established, it is reasonable to speculate that our cultured CD56^+^ DP-MSC populations are primarily composed of cells that possess greater capacities to differentiate into cells of the neuronal lineage. This hypothesis is strengthened by the fact that DP-MSCs share a common origin with peripheral nerve glial progenitor cells (Kaukua et al., 2014) and by the fact that the neural crest origin of DP-MSCs confers them a greater capacity to differentiate into neuronal cells than MSCs of mesodermal origin such as bone marrow or adipose tissue MSCs (Arthur et al., 2008; Degistirici et al., 2008; Huang et al., 2009; Alipour et al., 2010; Karaöz et al., 2011; Bonnamain et al., 2013; Gervois et al., 2015). CD56 was also related to myogenic stem cell differentiation into myoblasts, chondrocytes and osteoblasts (Sinanan et al., 2004; Crisan et al., 2008; Lecourt et al., 2010). The CD56^+^ cell population within human muscle-derived cells was found to be heterogeneous and composed of lineage-committed myogenic cells and multipotent cells (Sinanan et al., 2004; Lecourt et al., 2010). Similarly to our results, a few percents of freshly isolated muscle stem cells expressed CD56, whereas more than 70% expressed this marker after three passages (Lecourt et al., 2010). In our study, increase in the percentage of CD56^+^ DP-MSCs with passages led to a modification of the relative proportions of the cell populations in culture between P1 and P4. Indeed, the most abundant DP-MSC populations were, in decreasing order, CD146^+^CD56^+^, CD146^+^MSCA-1^+^, and MSCA-1^+^CD56^+^ at the end of P1, whereas CD146^+^CD56^+^, MSCA-1^+^CD56^+^, and MSCA-1^+^CD146^+^ cells were the most abundant ones at the end of P4. MSCA-1^+^CD146^+^ were recently identified in cultured DP-MSCs (Tomlinson et al., 2015), but their proportion was lower than that we found (≈3 vs. ≈8% in our study), possibly owing to the presence of serum in the culture medium of Tomlinson et al. (2015). Co-expression of CD56 and MSCA-1 markers was previously observed in DP-MSCs and bone marrow MSCs (Battula et al., 2009; Sobiesiak et al., 2010; Tomlinson et al., 2015), and it was associated with high clonogenicity and multipotency. Co-expression of CD56 and CD146 has been described as a factor promoting muscle cell differentiation (Lecourt et al., 2010; Bosch et al., 2012). Finally, since CD56 and CD146 are both cell adhesion molecules important for cell–cell contacts and cell propagation, their co-expression in neighboring cells would help recreate the appropriate environment of the stem niche *in vivo* which allows them for retaining their stemness and pluripotency.

In summary, the data reported in this study suggest that DP-MSC populations *in vivo* possess diverse and complex phenotypes. They also highlight the modifications of DP-MSC fate that occur during *in vitro* expansion in serum-free medium, leading to the progressive selection of possibly committed populations. Further studies are needed to determine whether co-expression of specific MSC markers confers DP cells specific properties that could be used for the regeneration of human tissues, including the dental pulp, with standardized cell-based medicinal products.

## Author contributions

All authors contributed to the conception and the design of the work, to the analysis and interpretation of the data, and to the drafting of the manuscript. MD and HF performed experiments. All authors approve the submitted final version. All authors agree to be accountable for all aspects of the work.

### Conflict of interest statement

The authors declare that the research was conducted in the absence of any commercial or financial relationships that could be construed as a potential conflict of interest. The reviewer CNA and handling Editor declared their shared affiliation, and the handling Editor states that the process nevertheless met the standards of a fair and objective review.
